# Metabolic Hydrogen Flows in Rumen Fermentation: Principles and Possibilities of Interventions

**DOI:** 10.3389/fmicb.2020.00589

**Published:** 2020-04-15

**Authors:** Emilio M. Ungerfeld

**Affiliations:** Laboratorio de Fermentación Ruminal, Instituto de Investigaciones Agropecuarias (INIA), Centro Regional Carillanca, Temuco, Chile

**Keywords:** rumen, hydrogen, redox, fermentation, microorganisms, metabolism, kinetics, thermodynamics

## Abstract

Rumen fermentation affects ruminants productivity and the environmental impact of ruminant production. The release to the atmosphere of methane produced in the rumen is a loss of energy and a cause of climate change, and the profile of volatile fatty acids produced in the rumen affects the post-absorptive metabolism of the host animal. Rumen fermentation is shaped by intracellular and intercellular flows of metabolic hydrogen centered on the production, interspecies transfer, and incorporation of dihydrogen into competing pathways. Factors that affect the growth of methanogens and the rate of feed fermentation impact dihydrogen concentration in the rumen, which in turn controls the balance between pathways that produce and incorporate metabolic hydrogen, determining methane production and the profile of volatile fatty acids. A basic kinetic model of competition for dihydrogen is presented, and possibilities for intervention to redirect metabolic hydrogen from methanogenesis toward alternative useful electron sinks are discussed. The flows of metabolic hydrogen toward nutritionally beneficial sinks could be enhanced by adding to the rumen fermentation electron acceptors or direct fed microbials. It is proposed to screen hydrogenotrophs for dihydrogen thresholds and affinities, as well as identifying and studying microorganisms that produce and utilize intercellular electron carriers other than dihydrogen. These approaches can allow identifying potential microbial additives to compete with methanogens for metabolic hydrogen. The combination of adequate microbial additives or electron acceptors with inhibitors of methanogenesis can be effective approaches to decrease methane production and simultaneously redirect metabolic hydrogen toward end products of fermentation with a nutritional value for the host animal. The design of strategies to redirect metabolic hydrogen from methane to other sinks should be based on knowledge of the physicochemical control of rumen fermentation pathways. The application of new –omics techniques together with classical biochemistry methods and mechanistic modeling can lead to exciting developments in the understanding and manipulation of the flows of metabolic hydrogen in rumen fermentation.

## Introduction

The complex microbial community that inhabits the rumen allows ruminants to digest and transform fibrous carbohydrates unavailable to humans into useful products such as meat, milk, wool and traction. Critical to the symbiosis between the rumen microbiota and the host animal is the anaerobic condition of the rumen, which prevents the complete oxidation of carbohydrates to carbon dioxide (CO_2_) and water. Instead, carbohydrates are incompletely oxidized to volatile fatty acids (VFA) and gases, with the host animal absorbing and utilizing the VFA as sources and precursors of energy, fat, glucose, and non-essential amino acids (Armstrong and Blaxter, 1957).

Rumen fermentation not only provides the ruminant with VFA. Part of the negative Gibbs energy change (Δ*G*) associated with fermentation is used by rumen microbes to generate ATP that can be utilized for microbial growth, active transport of substrates, and motility. Microbial growth produces microbial protein, which is the principal (Wallace et al., 1997) and most economical source of amino acids for ruminants. Rumen microorganisms can also synthesize water-soluble vitamins, which thus do not need to be included in most ruminants diets (Weiss, 2017).

A product of rumen fermentation is methane (CH_4_), which is a potent greenhouse gas when released to the atmosphere, and also a loss of energy for ruminants (Eckard et al., 2010; Martin et al., 2010). Through the formation of CH_4_ and the profile of VFA produced, rumen fermentation has important consequences for animal productivity and the environment. Understanding how rumen fermentation is controlled can help designing strategies to manipulate it in desired directions. Central to rumen metabolism are the dynamics of metabolic hydrogen ([H]) production and utilization. The idea of understanding rumen energy metabolism as [H] flows through different biochemical pathways is not new (Czerkawski, 1986; Hegarty and Gerdes, 1999). The objectives of this paper are to review and critically examine [H] flows as the unifying principle to understand rumen fermentation. In particular, this paper will discuss (i) The control of the VFA profile and CH_4_ production by dihydrogen (H_2_), (ii) The principles underlying the competition for H_2_, (iii) The potential inhibitory effects of H_2_ and other intercellular electron (e^–^) carriers on the rates of fermentation and digestion in the rumen, and (iv) The relationship between the flows of [H] and microbial growth. All of these aspects have implications to animal productivity and the environment mediated by ruminal and post-absorptive metabolism.

## Background

### Definitions

The terms in the following list are in some cases defined bearing in mind their main significance with regard to rumen energy metabolism, acknowledging that other aspects might be more important in other areas of science.

**Electron (e^–^)** – a negatively charged sub-atomic particle.

**Redox reaction** – a chemical reaction involving an exchange of one or more e^–^ between two chemical compounds, an e^–^ donor and an e^–^ acceptor.

**Reducing potential (*Eh*)** – a measure of the tendency of a chemical compound or a system to donate e^–^ under certain defined conditions.

**Proton (p^+^ or H^+^)** – a positively charged sub-atomic particle released by acids in aqueous solutions.

**Metabolic hydrogen ([H])** – the sum of all hydrogen atoms that can be exchanged between molecules in a living cell or a microbial ecosystem, or any other defined living system.

**Redox cofactors** – intracellular molecules that act as e^–^ acceptors and donors in redox reactions to transfer e^–^ between metabolic intermediates. Redox cofactors have, therefore, different oxidation stages, for example: reduced and oxidized nicotinamide adenine dinucleotide (NADH and NAD^+^, respectively), reduced and oxidized ferredoxin (Fd_red_^2^^–^ and Fd_ox_, respectively), reduced, semi-reduced and oxidized flavin mononucleotide (FMNH_2_, FMNH and FMN, respectively), reduced and oxidized flavin adenine dinucleotide (FADH_2_ and FAD, respectively), and reduced and oxidized tocopherols. Some cofactors accept and donate pairs of e^–^ (e.g., ferredoxin), others accept and donate pairs of hydrogen atoms (H = 1 e^–^ + 1 H^+^, e.g., FAD), or one hydrogen atom (e.g., tocopherols). The NADH/NAD^+^ pair accepts and donates two e^–^ per H^+^: NAD^+^ + [2H] ↔ NADH + H^+^, with [2H] = 2 H^+^ + 2 e^–^.

**Fermentation** – an incomplete oxidation in which the ultimate e^–^ acceptors are carbon compounds produced in the process itself.

**Metabolic hydrogen production (or release)** – the transfer of [H] from e^–^ donors that are metabolic intermediates to oxidized intracellular cofactors.

**Metabolic hydrogen incorporation** – the transfer of [H] from reduced intracellular cofactors to e^–^ acceptors intermediate in metabolism.

**Metabolic hydrogen sink (or electron sink)** – reduced end product of fermentation whose pathway of formation involves reactions that incorporate [H]. Note that some [H] sinks are not net [H] sinks in the sense that their production involves greater [H] production than incorporation, e.g., the production of butyrate from hexoses.

**Reducing equivalents pairs ([2H])** – a pair of hydrogen atoms, or a mole of hydrogen atom pairs, produced or incorporated in a metabolic reaction or pathway. This concept is used to quantify [H] transactions as [2H] production and [2H] incorporation.

**Hydrogenases** – enzymes that catalyze: (i) The formation of H_2_ from e^–^ donated by reduced intracellular cofactors (H_2_-evolving hydrogenases), and/or (ii) The reduction of an intracellular cofactor by H_2_ (H_2_-incorporating hydrogenases).

**Interspecies hydrogen transfer** – process in which H_2_ produced and released by a microbial cell is incorporated by another microbial cell. Interspecies H_2_ transfer decreases H_2_ concentration in the proximity of the H_2_-producing cell, thermodynamically favoring H_2_ production.

**Intercellular electron carriers** – reduced compounds intermediate of fermentation pathways that incorporate [H] in their formation, and are released by some microbial cells and taken up by others e.g., H_2_, formate, ethanol, lactate and succinate.

**Substrate level phosphorylation** – generation of ATP in which a phosphate group is donated by a phosphorylated organic compound to phosphorylate ADP to ATP.

**Electron transport-linked phosphorylation (ETLP)** – generation of ATP from ADP and phosphate driven by a transmembrane electrochemical gradient of H^+^ or sodium cations. The electrochemical gradient is created by the extrusion of H^+^ or sodium cations of the microbial cell, which is in turn coupled to an intracellular redox reaction.

### Carbohydrate Metabolism and Production of Volatile Fatty Acids

Living organisms can generate energy for anabolic functions through thermodynamically favorable flows of [H]. In anaerobic environments such as the rumen, the main e^–^ acceptors are carbon compounds generated in the fermentation process itself, with methanogenesis being the most important [H] disposal pathway. The main pathways of carbohydrate fermentation in the rumen have been reviewed and investigated by Russell and Wallace (1997), Russell (2002), and Hackmann et al. (2017), and the reader is referred to those scientific papers for detailed information. The focus in this subsection will be on reactions central to [H] transfer, and their implications with regard to the balance of [2H] production and incorporation of each fermentation pathway.

**FIGURE 1:**
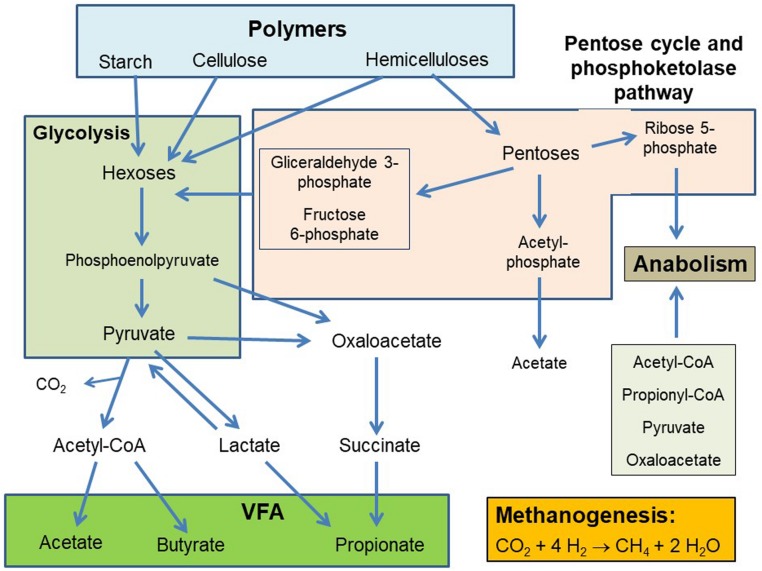
Simplified scheme of carbohydrates fermentation in the rumen.

It is understood that the mixed rumen microbiota metabolizes over 90% of hexoses (in turn released from the hydrolysis of complex carbohydrates such as cellulose and starch) to pyruvate through the glycolytic pathway. Pyruvate (and also phosphoenolpyruvate in propionate production) is a central branching point at which the different pathways leading to the formation of the three main VFA, acetate, propionate and butyrate, diverge (Russell and Wallace, 1997; Figure 1). Recently, Hackmann et al. (2017) examined complete genomes of various rumen bacteria that have been isolated and grown in pure culture, and found that several bacterial species encoded for incomplete glycolytic pathways as well as several alternative pathways of carbohydrate metabolism, such as the *Bifidobacterium* pathway.

Hemicelluloses are also abundant as plant structural carbohydrates, and are rich in pentoses such as xylose and arabinose (Scheller and Ulvskov, 2010). In the rumen, pentoses are metabolized through the pentose cycle and to a lesser extent through the transketolase cleavage (Russell and Wallace, 1997). This results in the production of glyceraldehyde-3-phosphate and fructose 6-phosphate, which can enter glycolysis, of acetyl-phosphate, which can be converted to acetate, and of ribose 5-phosphate, which can be used to synthesize nucleotides and histidine (Voet and Voet, 1995; Figure 1).

**FIGURE 2:**
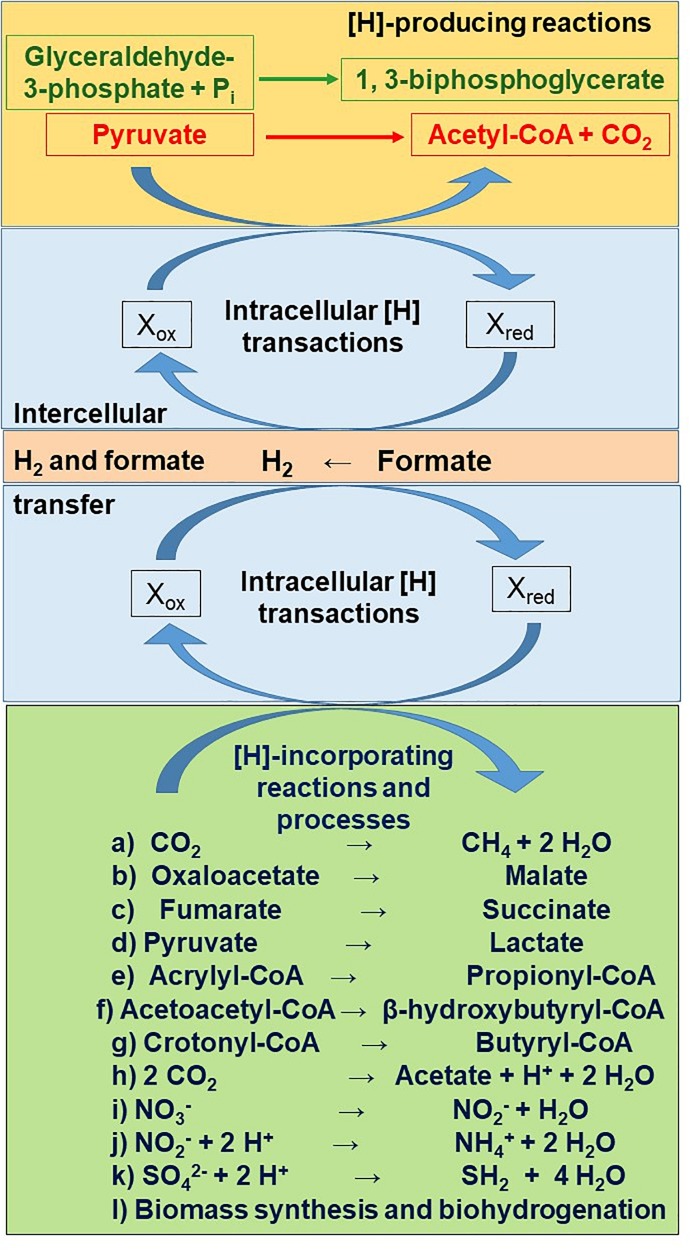
Main reactions releasing (orange rectangle) and incorporating (green rectangle) metabolic hydrogen ([H]), as connected by intracellular (sky blue rectangle) and intercellular (salmon rectangle) metabolic hydrogen transactions. The stoichiometry of production and incorporation of reducing equivalents is not depicted.

Glycolysis involves the oxidation of glyceraldehyde-3-phosphate to 1,3-biphosphoglycerate coupled to the reduction of NAD^+^ to NADH (Voet and Voet, 1995). Metabolic hydrogen is also produced in the first step of acetate and butyrate production, the oxidative decarboxylation of pyruvate to acetyl-CoA (Figure 2). Depending on the hydrogenase catalyzing pyruvate decarboxylation, [H] can reduce Fd_ox_ to Fd_red_^2^, or CO_2_ to formate (Russell and Wallace, 1997; Hegarty and Gerdes, 1999; Russell, 2002). Ferredoxins (see section “Definitions”) are iron sulfur proteins that act as e^–^ carriers through the reduction of one iron atom per iron sulfur cluster: 2 Fe^2+^ + 2 Fe^3+^ (oxidized form) + e^–^ → 3 Fe^2+^ + Fe^3+^ (reduced form) (Gottschalk, 1986).

For metabolism of carbohydrates to continue, reduced cofactors need to be re-oxidized (Wolin et al., 1997). Hydrogenases transfer e^–^ from reduced cofactors to H^+^, forming H_2_ (Frei, 2013; Figure 2). Dihydrogen does not accumulate in the rumen, as it is transferred from the rumen consortium of bacteria, protozoa and fungi to methanogens (Janssen, 2010). Hydrogenases also catalyze the uptake and incorporation of H_2_ by methanogens and other hydrogenotrophs (Frey, 2002; Søndergaard et al., 2016). Apart from H_2_, rumen methanogens and other hydrogenotrophs can also use as [H] donors other intercellular e^–^ carriers such as formate, methanol, ethanol and methylamines (Asanuma et al., 1999; St-Pierre et al., 2015; Patra et al., 2017).

Electrons in reduced cofactors or in H_2_ or formate can also be disposed through their incorporation into pathways other than methanogenesis (Figure 2). In the randomizing pathway of propionate formation (so called because carbon labeled in position 2 of pyruvate is randomized to positions 2 and 3 of succinate), [H] donated by H_2_, formate, NADH or lactate is incorporated in the reductions of oxaloacetate to malate and fumarate to succinate (Henderson, 1980; Gottschalk, 1986; Russell and Wallace, 1997; Asanuma et al., 1999). The reduction of fumarate to succinate is coupled to ETLP (De Vries et al., 1973; Gottschalk, 1986; Kröger et al., 2002). Succinate can be metabolized to propionate by the succinate producer itself or it can be transferred to succinate utilizers as an intercellular e^–^ carrier.

Lactate is an intermediate in the non-randomizing pathway of propionate formation (so called because carbon labeled in position 2 in pyruvate appears in position 2 in propionate). Lactate is formed from the reduction of pyruvate with [H] donated by NADH. Lactate can be intracellularly activated to lactyl-CoA, which is then dehydrated to acrylyl-CoA. Acrylyl-CoA is then reduced to propionyl-CoA with reduced flavoprotein (Gottschalk, 1986) or NADH (Hackmann et al., 2017) as [H] donor, in a reaction that has not been found to be coupled with ATP generation through ETLP (Thauer et al., 1977; Seeliger et al., 2002). Lactate can also be excreted and taken up by other microbial cells that convert it to acetate, propionate or butyrate (Chen et al., 2019).

**FIGURE 3:**
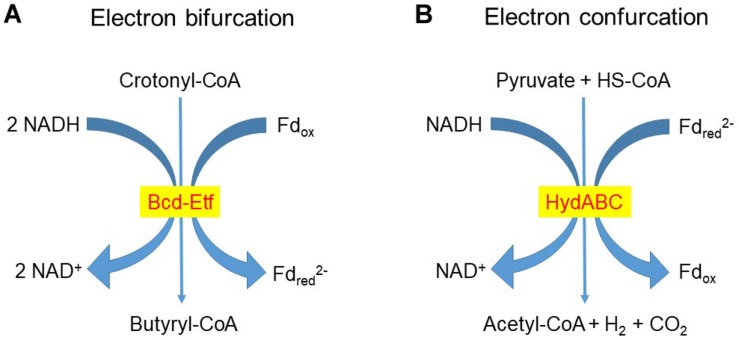
Examples of **(A)** Electron bifurcation and **(B)** Electron confurcation redox reactions. Electron bifurcation in the reduction of Fd_ox_ by NADH coupled to the reduction of crotonyl-CoA to butyryl-CoA catalyzed by Bcd-Etf was proposed by Hackmann and Firkins (2015) to operate in rumen butyrivibrios (genera *Butyrivibrio* and *Pseudobutyrivibrio*). In the rumen bacterium *R. albus* the electron-bifurcating hydrogenase HydABC catalyzes the formation of dihydrogen (H_2_) from NADH and reduced ferredoxin (Fd_red_^2^^–^) (Zheng et al., 2014; Buckel and Thauer, 2018b).

The conversion of two molecules of acetyl-CoA to butyrate also involves two [H]-incorporating steps, with NADH as the reductant in the conversion of acetoacetyl-CoA to β-hydroxybutyryl-CoA and of crotonyl-CoA to butyryl-CoA. In the reduction of crotonyl-CoA to butyryl-CoA, Fd_ox_ is simultaneously reduced by a second molecule of NADH to Fd_red_^2^^–^ in a process called electron bifurcation (Buckel and Thauer, 2013; Figure 3A; see also section “The Role of Dihydrogen as an Intercellular Electron Carrier”). The oxidation of Fd_red_^2^^–^ so formed by electron bifurcation can result in H^+^ extrusion and ATP generation by ETLP (Hackmann and Firkins, 2015; Hackmann et al., 2017).

### The Role of Dihydrogen as an Intercellular Electron Carrier

Dihydrogen has a central role in the flows of [H] in the rumen. Genes encoding hydrogenases are widespread in the genomes of rumen bacteria and archaea, highlighting that an important proportion of [H] is transferred and incorporated between cells as H_2_ (Greening et al., 2019). This agrees with the historical finding by Hungate (1967) of H_2_ being the main [H] donor for CH_4_ formation in rumen fermentation.

Interspecies H_2_ transfer thermodynamically favors the re-oxidation of intracellular cofactors as the H_2_-consuming microorganisms help decreasing H_2_ concentration (Wolin et al., 1997; Lubner and Peters, 2017). In the presence of methanogens, or propionate or succinate producers, H_2_ producers shifted fermentation away from formate, lactate and ethanol toward acetate, and cellulose digestion increased. Production of H_2_ was stimulated as H_2_ accumulation was relieved in the presence of methanogens or other hydrogenotrophs (Chen and Wolin, 1977; Bauchop and Mountfort, 1981; Marvin-Sikkema et al., 1990; Wolin et al., 1997).

The participation of ferredoxins is fundamental in H_2_ formation and incorporation. Ferredoxins have very low standard reducing potentials comparable to the H_2_/2H^+^ couple, which allows them to donate e^–^ to a hydrogenase to reduce H^+^ to H_2_ (Gottschalk, 1986). In microbial cells ferredoxins are typically more than 90% reduced, which makes them strong e^–^ donors. Reduction of Fd_ox_ is thus thermodynamically very unfavorable, and it occurs through flavin-based electron bifurcation, with the donation of e^–^ to Fd_ox_ coupled to a stoichiometrical donation of e^–^ by the same e^–^ donor to a strong e^–^ acceptor. In this way Fd_ox_ can be reduced by NADH (Figure 3A) or by H_2_ in bacteria and archaea. In turn, the re-oxidation of Fd_red_^2^^–^ can be coupled to energy conservation through the generation of a transmembrane electrochemical gradient. Some of the coupled reductions that can drive the reduction of Fd_ox_ are crotonyl-CoA to butyryl-CoA with NADH as the e^–^ donor, NAD^+^ to NADH with H_2_ or NADPH as e^–^ donors, NADP^+^ to NADPH with H_2_ as e^–^ donor, pyruvate to lactate with NADH as e^–^ donor, and CO_2_ to formate with NADPH as e^–^ donor. In methanogens, the high potential e^–^ acceptor involved in electron bifurcation in the last step of methanogenesis reaction is the CoM-S-S-CoB heterodisulfide that is split into HS-CoM and HS-CoB by the e^–^ donor F_420_H_2_ (reduced 8-hydroxy-5-deazaflavin). The reverse reaction of bifurcation, in which H_2_ can be produced, is called confurcation (Figure 3B; Nitschke and Russell, 2012; Buckel and Thauer, 2013, 2018a,b).

Genes encoding confurcating hydrogenases that oxidize NADH and Fd_red_^2^^–^ to H_2_ were the most abundant hydrogenases-encoding genes from 501 rumen bacteria genomes from the Hungate culture collection and others. Furthermore, confurcating hydrogenases were the most abundant hydrogenase transcripts in sheep rumens, which shows the importance of this relatively recently discovered e^–^ transfer mechanism in rumen fermentation (Greening et al., 2019). The rumen bacterium *Ruminococcus albus*, for example, can produce H_2_ from NADH in confurcation with the oxidation of Fd_red_^2^^–^ (Zheng et al., 2014). Greening et al. (2019) found that the expression in *R. albus* of an 8-gene cluster encoding for an alcohol and aldehyde dehydrogenase involved in the production of ethanol, a ferredoxin H_2_-evolving hydrogenase, and a sensory hydrogenase, were sharply decreased in co-culture with the H_2_-utilizer *Wolinella succinogenes* in comparison to the mono-culture. In the co-culture, NADH was re-oxidized only through confurcation with Fd_red_^2^^–^, as NADH was not utilized as reductant for ethanol production.

### Production of Volatile Fatty Acids and Balances of Reducing Equivalents

The [H]-producing and -incorporating reactions in the different fermentation pathways result in different stoichiometries of [2H] production and incorporation per mole of VFA produced, which can be used to calculate [2H] balances (Marty and Demeyer, 1973). Acetate, and to a lesser extent, butyrate production from glucose, are associated with the net production of [2H]. On the other hand, propionate production implies a net incorporation of [2H]. Thus, propionate competes with CH_4_ as a [H] sink in rumen fermentation whereas acetate and butyrate formation release [H] that can be utilized by methanogens to reduce CO_2_ to CH_4_ (Janssen, 2010). The formation of CH_4_ in rumen fermentation is thus closely associated to the profile of VFA formed.

*In vitro* balances of [2H] production and incorporation show that CH_4_ is the main sink of [H] in the rumen fermentation with functional methanogenesis (Ungerfeld, 2015b). Strictly speaking, it is unknown if CH_4_ is the main [H] sink in the live animal, because there are no *in vivo* experimental reports in which the production of both VFA and gases has been simultaneously measured. However, estimations based on the Cabezas-Garcia et al. (2017) meta-analysis suggest that CH_4_ is also almost surely the most important [H] sink in rumen fermentation in dairy cows on mixed diets (calculation not shown). Furthermore, the importance of CH_4_ as [H] sink agrees with the abundance in the rumen of sheep of archaeal hydrogenases and reductases (Snelling and Wallace, 2017) and their transcripts (Greening et al., 2019).

The recent discovery that many rumen bacteria encode an incomplete glycolytic pathways and alternative pathways of hexose metabolism (Hackmann et al., 2017) can potentially add complexity and unknown stoichiometries of [2H] production and incorporation associated to the production of each VFA. The formation of glyceraldehyde-3-phosphate in the oxidative pentose phosphate pathway would greatly augment [2H] produced in the conversion of glucose to pyruvate from 2 to 14 [2H] mole/mole glucose. Conversely, acetate production through the *Bifidobacterium* pathway is not associated to [2H] production or incorporation, in contrast to 4 × [2H] mole/mole glucose if produced through glycolysis or the phosphoketolase pathway. The flows of carbon through different pathways depend on the genetic makeup of the rumen microbial community (i.e., pathways encoded), the abundance of the different microbial populations encoding each pathway, gene expression in the different microorganisms, enzyme and substrate kinetics, and thermodynamic feasibility of reactions.

### Other Incorporating Pathways Incorporating Metabolic Hydrogen

The presence of genes and transcripts of hydrogenases catalyzing H_2_ uptake in nitrate and sulfate reduction and reductive acetogenesis, has been reported in sheep rumens (Greening et al., 2019). The reduction of nitrate and sulfate thermodynamically outcompetes methanogenesis (Ungerfeld and Kohn, 2006). However, the concentration of these e^–^ acceptors in the rumen usually limits the rate of [H] incorporation in their reduction, unless they are supplemented to the diet as salts (Van Zijderveld et al., 2010). Nitrate can also be naturally present at high contents in grasses, which can lead to toxicity caused by the absorption and passage to blood of its reduction intermediate nitrite (McKenzie et al., 2004). Reductive acetogenesis, the reduction of CO_2_ with H_2_ to acetate, seems to be thermodynamically outcompeted by methanogenesis in the rumen (Kohn and Boston, 2000), although it is functional or even predominates over methanogenesis in the gastrointestinal tract of some termites, cockroaches, kangaroos, pre-ruminant lambs, rodents, pigs, and some humans (Joblin, 1999; Gagen et al., 2012; Klieve et al., 2012). Some reductive acetogens inhabit the rumen, but as they are not obligate hydrogenotrophs it is possible that they survive mainly by metabolizing carbohydrates (Joblin, 1999). That said, a recent study found that reductive acetogenesis was a minor, but not insignificant, [H] sink in the rumen (Raju, 2016). The presence of genes (Denman et al., 2015) and transcripts (Greening et al., 2019) of hydrogenases involved in reductive acetogenesis has also been reported.

### Importance of the Rumen Fermentation Profile

The profile of products formed in rumen fermentation has implications for animal productivity and the environment. Despite its importance as the main [H] sink in rumen fermentation, the release of CH_4_ to the atmosphere represents an energy loss ranging between 2 and 12% of ingested gross energy (Johnson and Johnson, 1995) and was identified early on in ruminant nutrition research as an energy inefficiency in rumen fermentation and an opportunity to improve animal productivity (Czerkawski and Breckenridge, 1975a; Davies et al., 1982). More recently, increasing concerns about climate change have raised interest in the abatement of CH_4_ emissions from ruminants. Emissions of enteric CH_4_ are estimated to account for about 6% of total anthropogenic emissions of greenhouse gases expressed as CO_2_-eq i.e., the sum of the emissions of each greenhouse gas weighted by its global warming potential (Gerber et al., 2013).

The profile of VFA absorbed from the rumen has also consequences on the host animal’s post-absorptive metabolism (Ungerfeld, 2013). Inhibiting methanogenesis can shift fermentation toward propionate production (Janssen, 2010), which is the main glucose precursor in ruminants (Aschenbach et al., 2010). Increased propionate production can be important to animals with high requirements for glucose such as high producing dairy cows in early lactation. On the other hand, propionate is a satiety signal in ruminants and can decrease feed intake (Allen et al., 2009) and milk fat content (Maxin et al., 2011). In turn, an increased supply of acetate increases milk fat percentage (Sheperd and Combs, 1998; Maxin et al., 2011; Urrutia et al., 2019).

## Discussion

### The Control of the Rumen Fermentation Profile

Diets that are rich in fiber produce a profile of VFA high in acetate and low in propionate, with a relatively high production of CH_4_ per unit of digested organic matter. On the other hand, concentrates, which are richer in starch, are fermented to more propionate and less CH_4_ (Johnson and Johnson, 1995; Janssen, 2010; Ungerfeld, 2013). The diet effect on the acetate to propionate ratio cannot be explained simply by different chemical composition of cellulose and starch, as they are both hydrolyzed to glucose (Janssen, 2010).

Janssen (2010) proposed a mechanism based on methanogens growth rate and the resulting H_2_ concentration, to explain how concentrates shift rumen fermentation from acetate to propionate and lower CH_4_ production. The replacement of roughages with concentrates induces changes such as increased rumen outflow rates and lower rumen pH. High rumen outflow rates impose methanogens that are not washed out of the rumen faster growth rates. Based on the Monod relationship of microbial growth, H_2_ concentration must increase when methanogens grow faster. In turn, greater H_2_ concentration would thermodynamically inhibit H_2_ production, and by doing so also inhibit acetate production, which is associated with H_2_ production. Greater H_2_ concentration would conversely favor [H] redirection toward alternative [H] sinks such as propionate. In agreement, the concentration of dissolved H_2_ in the rumen has been reported to associate negatively with acetate and positively with propionate molar percentages (Wang et al., 2017, 2018), although an association with propionate molar percentage was not observed in another study (Wang et al., 2016).

Similarly, methanogens are sensitive to the low rumen pH induced by feeding concentrates. A decrease in rumen pH is expected to decrease methanogens maximum growth rates, and, according to the Monod relationship, H_2_ concentration would increase if methanogens growth rate is maintained. An increase in H_2_ concentration would again thermodynamically shift fermentation from acetate to propionate. A similar explanation was provided for the accumulation of H_2_ and shift from acetate to propionate caused by chemical inhibitors of methanogenesis (Janssen, 2010).

Several experiments with defined cultures comparing the fermentation profile of pure cultures of H_2_ producers with co-cultures of the same organisms growing with methanogens demonstrate the profound influence of H_2_ removal by the methanogen on the fermentation profile of the H_2_-producing microorganism (Wolin et al., 1997). These insightful experiments provide a simple proof of concept for the theory proposed by Janssen (2010): in the absence of methanogens in the mono-culture (i.e., an analogous situation to methanogens completely washed out because a very high rumen outflow rate, or completely inhibited by low pH or an inhibitor of methanogenesis), H_2_ accumulates, inhibiting H_2_ formation and decreasing acetate production (i.e., a H_2_-releasing pathway). This in turn directs [H] toward reduced intermediates of rumen fermentation such as formate, ethanol, lactate and/or succinate (Chung, 1976; Chen and Wolin, 1977; Bauchop and Mountfort, 1981; Marvin-Sikkema et al., 1990; Pavlostathis et al., 1990), and [H] sinks such as propionate (Chen and Wolin, 1977) or butyrate (Chung, 1976). When H_2_ producers were co-cultured with methanogens they greatly diminished or stopped producing formate, ethanol, lactate and/or succinate, as well as propionate. The co-cultures accumulated less H_2_, and as CH_4_ formation removed H_2_, lower H_2_ concentration thermodynamically favored acetate production.

Increasing outflow rates in some continuous culture experiments (Isaacson et al., 1975; Stanier and Davies, 1981) agree with the predictions of the Janssen (2010) model. Results of the experiment by Wenner et al. (2017) do not fully agree, as, contrary to the model expectations decreasing pH decreased H_2_ concentration.

Immediately after feed ingestion, the most readily digestible feed components are rapidly digested and fermented, and H_2_ concentration rises (Robinson et al., 1981; Janssen, 2010; Guyader et al., 2015). After feed ingestion, increases in H_2_ emissions have been shown to occur earlier and faster than increases in CH_4_ (Rooke et al., 2014; Van Lingen et al., 2017; Söllinger et al., 2018), although this pattern has not occurred in all studies (Hillman et al., 1985). The peak in H_2_ emission preceding the evolution of CH_4_ production has been modeled by Van Lingen et al. (2019), and interpreted by Rooke et al. (2014) as the consequence of rapid fermentation and H_2_ production exceeding the capacity of methanogens to utilize all the H_2_ produced.

The lag period between the CH_4_ and H_2_ peaks observed in some studies suggests that, when fermentation is rapid, methanogens growth and/or the expression of genes encoding for methanogenesis enzymes lags behind rapid H_2_ evolution. In that regard, Söllinger et al. (2018) reported that whereas archaeal 16S rRNA genes abundance peaked at 1 h after feeding, methanogenesis mRNA abundance did not peak until 3 h after feeding. The question becomes what impedes or retards methanogens to respond with more rapid gene expression to make use of the elevated H_2_ concentrations occurring after a meal. It is possible that temporal increases in outflow rates occurring after feeding episodes (Van Lingen et al., 2019), result in high H_2_ concentration by increasing methanogens growth rate, as proposed by Janssen (2010). However, increasing the concentration of glucose in a chemostat at a constant pH and outflow rate still decreased CH_4_ production per mole of glucose fermented (Isaacson et al., 1975), which suggests a limitation of methanogenesis independent of outflow rate or pH to use all of the H_2_ made available by the rapid fermentation of glucose at high concentration (although H_2_ was not measured in that experiment). In 48 h batch cultures, there were distinct effects of pH and substrate composition (hay or cracked corn) on H_2_, CH_4_ and the acetate to propionate ratio (Russell, 1998), which can also be interpreted as an indication of an effect of the rate of fermentation *per se* independent of outflow rates or pH. It has also been speculated that the evolution of rumen H_2_-incorporating hydrogenases in the rumen environment with low H_2_ concentration may have resulted in low *K*_m_ but also low *V*_max_ for H_2_ (Ungerfeld, 2015a). This idea, however, does not agree with the high frequency of genomes of rumen organisms encoding [FeFe]-hydrogenases, and the high abundance of transcripts of various types of [FeFe]-hydrogenases in sheep rumens (Greening et al., 2019), as [FeFe]-hydrogenases have higher *V*_max_ and *K*_m_ for H_2_ uptake than [NiFe] hydrogenases (Frey, 2002).

### Effects of Electron Carriers Other Than Dihydrogen on the Rumen Fermentation Profile

Eighteen percent of rumen CH_4_ was estimated to be produced from formate as [H] donor (Hungate et al., 1970), and formate can be the [H] donor in fumarate reduction to succinate (Asanuma et al., 1999). It is thus possible that, the same as H_2_, formate concentration has an influence on CH_4_ production and the VFA profile. Other than an accumulation of formate as a response to methanogenesis inhibitors in some studies (Ungerfeld et al., 2003; Martinez-Fernandez et al., 2016, 2017), the effects of variables such as outflow rates, pH, or rate of fermentation on formate concentration, have not been investigated to the author’s knowledge. Generating information about the relationship between those variables and formate concentration, and how they relate to methanogens growth rate would be important for evaluating the influence of formate on the VFA profile, and integrating formate to a model of [H] flows in rumen fermentation.

Lactate is another intercellular e^–^ carrier which, except for lactic acidosis, normally does not accumulate in the rumen and is extensively converted to VFA by various lactate utilizers (Chen et al., 2019). Small amounts of lactate have been reported to accumulate as a consequence of inhibiting methanogenesis in some (Amgarten et al., 1981), but not all (Božic et al., 2009; Martinez-Fernandez et al., 2016), studies. In general, lactate accumulation in the rumen is the result of lactate production rate surpassing lactate utilization as a consequence of rapid fermentation. A possible enhancement in the role of lactate as an intermediate of butyrate production in low CH_4_-producing sheep (Kamke et al., 2016) deserves further study (see section “The Competition for Dihydrogen”).

Succinate concentration in the rumen is typically low as it is rapidly converted to propionate (Blackburn and Hungate, 1963; Immig, 1996). It thus seems that succinate concentration exerts little influence on CH_4_ production and the VFA profile, although the finding by Kamke et al. (2016) of greater abundance of genes involved in the conversion of succinate to butyrate in low CH_4_-producing sheep prompts for more investigation.

### The Competition for Dihydrogen

The principle proposed by Janssen (2010) relating rumen H_2_ concentration to methanogens growth rates could be in theory extended to other hydrogenotrophs, provided that their pathway of H_2_ incorporation is thermodynamically feasible. The rate of H_2_ uptake by a methanogen would follow a Michaelis-Menten kinetics-wise function:

(1) $$vmet=\frac{Vmaxmet\left[\text{H}2\right]}{\left(Kmmet+\left[\text{H}2\right]\right)}$$

where *v*_met_ is the rate of H_2_ uptake (e.g., mol L^–1^ min^–1^), *V*_max met_ is the maximum rate of H_2_ uptake with non-limiting H_2_ concentration, [H_2_] is the concentration of H_2_, and *K*_m met_ is the apparent affinity for H_2_ i.e., the concentration of H_2_ at which the rate of H_2_ uptake is half maximal.

If a methanogen was growing in co-culture with another hydrogenotroph whose rate of H_2_ uptake was limited by H_2_ concentration, and H_2_ concentration was above the H_2_ thresholds (Cord-Ruwisch et al., 1988) of both organisms, it can be deduced (Supplementary Appendix S1) that the proportion of total H_2_ uptake incorporated into methanogenesis would be equal to:

(2) $$\begin{array}{c} \frac{vmet}{\left(vmet+valt\right)}\\ =\frac{Vmaxmet\left(Kmalt+\left[\text{H}2\right]\right)}{\left[Vmaxmet\left(Kmalt+\left[\text{H}2\right]\right)+Vmaxalt\left(Kmmet+\left[\text{H}2\right]\right)\right]} \end{array}$$

where *v*_alt_ and *V*_maxalt_ are the rate and the maximum rate of H_2_ uptake of the alternative hydrogenotroph, respectively, and *K*_m alt_ is the affinity for H_2_ of the alternative hydrogenotroph, with the rest of the variables defined as in Eq. 1. This equation does not take into account possible thermodynamic constraints and differences in the efficiency of microbial growth.

**FIGURE 4:**
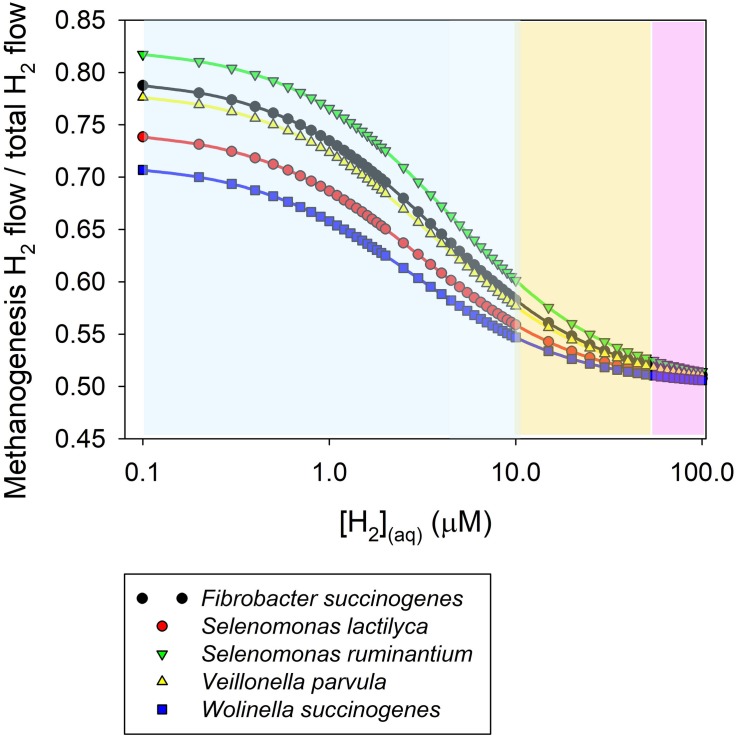
Simulation of the proportion of dihydrogen taken up by methanogens in co-culture with various fumarate reducers as a function of dissolved H_2_ concentration. The simulation was conducted based on a kinetic Michaelis-Menten-wise competition for dihydrogen. Apparent *K*_m_ for H_2_ uptake were reported by Asanuma et al. (1999). An equal *V*_max_ for dihydrogen uptake is assumed. The range of dissolved H_2_ concentration is based on Table 1. The sky blue area corresponds approximately to baseline dissolved H_2_ concentrations (i.e., in between meals). The salmon area corresponds approximately to H_2_ concentration peaks occurring closely after feeding. The purple area corresponds approximately to the range of H_2_ concentration that could be observed when methanogenesis is inhibited.

Figure 4 shows a simulation of the proportion of H_2_ uptake incorporated into methanogenesis as a function of H_2_ concentration according to Eq. 2 in co-cultures of mixed methanogens and various hydrogenotrophs that reduce fumarate to succinate. The *K*_m_ values for methanogens and fumarate reducers used in the simulation were reported by Asanuma et al. (1999). An equal *V*_max_ was assumed for methanogenesis and fumarate reduction in this simulation. The range of H_2_ concentration in Figure 4 is based on dissolved H_2_ concentration measured directly in various *in vivo* studies (Table 1). It can be seen in Figure 4 that as H_2_ concentration increases, the proportion of H_2_ taken up by methanogens decrease and approaches 1/2 (Supplementary Appendix S2). It can be shown that if the *V*_max_ of the alternative hydrogenotroph doubled the *V*_max_ of the methanogens, the proportion of H_2_ taken up by methanogens would trend to 1/3 as H_2_ concentration increases (Supplementary Appendix S3).

**TABLE 1:** Dissolved dihydrogen concentration in the rumen.

References	Treatment or condition	Method of measurement	H_2_ (μM)^1^
Hungate (1967)	Non-inhibited methanogenesis	H_2_ extraction procedure	0.19–30.4
Robinson et al. (1981)	Non-inhibited methanogenesis	H_2_ extraction procedure	2–15
Hillman et al. (1985)	Non-inhibited methanogenesis	Mass spectrometry	0.6–5.8
Smolenski and Robinson (1988)	Non-inhibited methanogenesis	H_2_ sensor	0.36–20.1
Guyader et al. (2015)	Non-inhibited methanogenesis	H_2_ sensor	3.58
	Nitrate		45.3
	Linseed		4.03
	Nitrate + linseed		21
Wang et al. (2016)	Oat grass	H_2_ extraction procedure	6.49
	Barley straw		2.34
Wang et al. (2017)	Control	H_2_ extraction procedure	1.02
	H_2_ released with Mg		1.99
Wang et al. (2018)	Control	H_2_ extraction procedure	2.37
	Nitrate		4.79
Ma et al. (2019)	Control	H_2_ extraction procedure	1.76
	H_2_ released with Mg		2.68
Melgar et al. (2019)	Control	Gas-stripping	7.3
	Methanogenesis inhibited with 3-nitrooxypropanol		43.6

*^1^Greater ranges in H_2_ concentration in some studies in which methanogenesis was not inhibited are due to multiple measurements throughout the day in animals fed once or twice a day, rather than to variation caused by treatments imposed.*

For *n* hydrogenotrophs (including *m* methanogens), the proportion of total H_2_ taken up by *m* methanogens, can be generalized to:

$$\frac{\sum_{j=1}^{j=m}vmetj}{\sum_{i=1}^{i=n}vi}=\frac{\prod_{i=1}^{i=n}\left(Kmi+\left[\text{H}2\right]\right)\sum_{j=1}^{j=m}\frac{vmaxmetj}{\left(Kmmetj+\left[\text{H}2\right]\right)}}{\sum_{i=1}^{i=n}vmaxi\left(Kmi+\left[\text{H}2\right]\right)}$$

(3, Supplementary Appendix S4)

At the low baseline H_2_ concentration prevailing in the rumen (Hungate, 1967), a low *K*_m_ for H_2_ incorporation is key in the competition for H_2_ among thermodynamically feasible H_2_-incorporating processes. Methanogens have a lower *K*_m_ for H_2_ than the fumarate reducers depicted in Figure 4, and consequently they would incorporate most of the H_2_ at the low H_2_ concentrations occurring between episodes of feed ingestion. Other *K*_m_ values reported for methanogens are shown in Table 2, and are similar the *K*_m_ of methanogens reported by Asanuma et al. (1999). In agreement with the predictions of Figure 4, previous co-culture experiments also show that the production of succinate or propionate by *Ruminococcus flavefaciens* growing on cellulose (Latham and Wolin, 1977) or by *Selenomonas ruminantium* growing on glucose or lactate (Chen and Wolin, 1977), decreased in the presence of methanogens compared to the pure cultures, although it continued being thermodynamically feasible, as it did not stop. In those co-culture experiments, H_2_ concentration (although it was not reported) was kept low by the methanogen, likely situating at the low end of the range of H_2_ concentration in Figure 4.

**TABLE 2:** Apparent *K*_m_ for dihydrogen of methanogens and fumarate reducers.

References	Microorganism	*K*_m_ (μM)
Hungate et al. (1970)	Methanogenesis by a mixed rumen culture	1
Hungate et al. (1970)	*Methanobrevibacter ruminantium*	1
Pavlostathis et al. (1990)	*Methanobrevibacter smithii*	1
Asanuma et al. (1999)	Mixed rumen methanogens	1.6
Asanuma et al. (1999)	*Fibrobacter succinogenes*	6.2
Asanuma et al. (1999)	*Selenomonas ruminantium*	7.5
Asanuma et al. (1999)	*Selenomonas lactylica*	4.7
Asanuma et al. (1999)	*Veillonella parvula*	5.8
Asanuma et al. (1999)	*Wolinella succinogenes*	4.0

As H_2_ concentration increases, as it occurs after feed ingestion, or when feeding concentrates, or if methanogenesis is inhibited, the *K*_m_ starts becoming less important to determine the partition of H_2_ incorporated into competing pathways, and a greater proportion of H_2_ would be incorporated into fumarate reduction to succinate (Figure 4). When H_2_ concentration is relatively high, a high *V*_max_ for H_2_ can potentially become very important to determine the flow of H_2_ incorporated by a certain microorganism in a particular pathway. If the *V*_max_ is expressed as the flow of H_2_ incorporated per gram of cell DM or cell protein, rather than the flow of H_2_ incorporated per volume of culture (or rumen contents), the flow of H_2_ into each pathway in the system will also depend on the cell density of each microbial species.

The incorporation of [H] into pathways alternative to methanogenesis can be limited by enzyme or substrate kinetics, or thermodynamics (Ungerfeld, 2015a). The addition of an e^–^ acceptor that can be metabolized to VFA can help removing substrate kinetics or thermodynamic constraints. In general, adding to rumen fermentation carboxylic acids that are propionate or butyrate precursors as e^–^ acceptors has had small effects on CH_4_ production *in vitro* (Callaway and Martin, 1996; Carro and Ranilla, 2003; Ungerfeld et al., 2003; Newbold et al., 2005; Riede et al., 2013) or *in vivo* (McGinn et al., 2004; Beauchemin and McGinn, 2006; Kolver and Aspin, 2006; Yang et al., 2012), although larger decreases in CH_4_ were observed in some experiments (Li et al., 2009; Wood et al., 2009). A likely interpretation is that much of the added propionate and butyrate precursor was not metabolized to the expected final product, and thus little [H] was directed away from CH_4_ formation (Carro and Ungerfeld, 2015). When methanogenesis was simultaneously inhibited with a chemical compound, increased availability of [H] not incorporated into CH_4_ favored the conversion of the added carboxylic acids to the expected end products. In the presence of inhibitors of methanogenesis, the addition of propionate precursors malate (Mohammed et al., 2004) or fumarate (Tatsuoka et al., 2008; Ebrahimi et al., 2011) increased propionate production *in vitro* and decreased H_2_ accumulation. In contrast, butyrate precursors did not decrease H_2_ accumulation caused by three inhibitors of methanogenesis in batch cultures (Ungerfeld et al., 2006). Martinez-Fernandez et al. (2017) successfully used phlorglucinol as an e^–^ acceptor to decrease the accumulation of H_2_ and formate in the rumen of steers whose methanogenesis was inhibited with chloroform. An increase in acetate concentration observed when phlorglucinol was supplemented agrees with previous studies which had shown that phlorglucinol was reduced to acetate by rumen microorganisms using H_2_ or formate as e^–^ donors (Martinez-Fernandez et al., 2017).

Microbial additives can help removing constraints to the incorporation of [H] into pathways alternative to methanogenesis whose rate is enzyme-limited. Jeyanathan et al. (2014) reviewed the use of direct-fed microbials to manipulate rumen biochemical pathways to decrease CH_4_ emissions. They proposed two main avenues to decrease CH_4_ formation in the rumen through the use of microbial additives: (i) Microbial additives that incorporate H_2_ into pathways alternative to methanogenesis, and (ii) Microbial additives that do not produce H_2_ in fermentation.

Microbial additives that compete with methanogens for H_2_ could be dosed into the rumen (Jeyanathan et al., 2014). It may also be possible to stimulate native rumen non-methanogenic hydrogenotrophs. Some fumarate reducers (Asanuma et al., 1999) and reductive acetogens (Chaucheyras et al., 1995; Joblin, 1999) were able to decrease CH_4_ production when grown in co-culture with methanogens, but because those experiments were conducted with elevated headspace H_2_, an ability of those organisms to compete for H_2_ at low concentration cannot be demonstrated (Figure 4). Methanogens would be expected to prevail over reductive acetogens in a co-culture at low H_2_ concentration due to their lower H_2_ thresholds (Cord-Ruwisch et al., 1988). Boccazzi and Patterson (2011) isolated rumen reductive acetogens with lower H_2_ thresholds than other reductive acetogens previously isolated from the rumen and with similar H_2_ thresholds to reductive acetogens from other environments. Yet, they still had higher H_2_ thresholds compared to methanogens (Table 3).

**TABLE 3:** Dihydrogen thresholds of methanogens and reductive acetogens from the rumen and other environments.

Microorganism	Environment	H_2_ threshold (ppm)	References
**Methanogens**			
*Methanospirillum hungatei*	Sewage sludge	30	Cord-Ruwisch et al. (1988)
*Methanobrevibacter smithii*	Primary sewage digester	100	Cord-Ruwisch et al. (1988)
*Methanobrevibacter arboriphilus*	Digested sewage sludge	90	Cord-Ruwisch et al. (1988)
*Methanobacterium formicicum*	Anaerobic sewage sludge digester	28	Cord-Ruwisch et al. (1988)
*Methanococcus vannielii*	Marine mud	75	Cord-Ruwisch et al. (1988)
Isolate 10-16B	Rumen	126	Le Van et al. (1998)
Isolate NI4A	Rumen	90 - 92	Boccazzi and Patterson (2011)
**Reductive acetogens**			
*Sporomusa termitida*	Termite hindgut	∼800	Breznak et al. (1988)
*Sporomusa termitida*	Termite hindgut	830	Cord-Ruwisch et al. (1988)
*Acetobacterium woodii* NZ Va 16	Not provided	520	Cord-Ruwisch et al. (1988)
*Acetobacterium carbinolicum*	Freshwater mud	950	Cord-Ruwisch et al. (1988)
*Acetitomaculum ruminis* 190A4	Rumen	3830	Le Van et al. (1998)
Two reductive acetogenic isolates	Rumen	∼750	Joblin (1999)
Isolate A2	Rumen	1383–2516	Boccazzi and Patterson (2011)
Isolate A4	Rumen	8007	Boccazzi and Patterson (2011)
Isolate A9	Rumen	1619–66157	Boccazzi and Patterson (2011)
Isolate A10	Rumen	208–1284	Boccazzi and Patterson (2011)
Isolate H3HH	Rumen	1390	Boccazzi and Patterson (2011)

Supplementation of rumen batch cultures with succinate and propionate producers caused mild to moderate decreases in CH_4_ production (Alazzeh et al., 2012; Mamuad et al., 2014). In another study, supplementing rumen batch cultures with fumarate-reducing enterococci caused large decreases in CH_4_ and increases in propionate concentration (Kim et al., 2016). A slight decrease in CH_4_ production per kilogram of ingested feed occurred when supplementing *Propionibacterium* strains to heifers fed a high-forage (Vyas et al., 2014a), but not a mixed (Vyas et al., 2016), or a high-concentrate (Vyas et al., 2014b), diet. Rumen succinate producers *W. succinogenes* and *Mannheimia succiniciproducens* could be interesting candidates to compete with methanogens at low H_2_ concentrations, as they possess [NiFe]-hydrogenases for H_2_ uptake (Søndergaard et al., 2016). [NiFe]-hydrogenases have *K*_m_ for H_2_ about two orders of magnitude lower than [FeFe] hydrogenases (Frey, 2002). However, the apparent *K*_m_ of *W. succinogenes* for H_2_ was still higher than that of methanogens (Asanuma et al., 1999; Table 2). *M. succiniciproducens* has been genetically engineered to improve its yield of succinate from glucose (Lee et al., 2006; Choi et al., 2016), which could help increasing its *V*_max_ for H_2_ uptake.

Nitrate reduction is thermodynamically more favorable than methanogenesis but may result in accumulation of the toxic intermediate nitrite. The addition of nitrite reducers may help avoiding nitrite toxicity while decreasing CH_4_ production (Jeyanathan et al., 2014). Nitrate should replace other sources of nitrogen on an isonitrogenous basis to avoid increasing the elimination of nitrogen in urine and the formation of nitrous oxide in the rumen, which is another potent greenhouse gas (Petersen et al., 2015). Sulfate reduction can also thermodynamically outcompete methanogenesis, although it generates the toxic reduced end product hydrogen sulfide (Jeyanathan et al., 2014).

Jeyanathan et al. (2014) also proposed that, by avoiding the formation of H_2_, the combined use of added lactate producers and the lactate utilizer *Megasphaera elsdenii* could channel [H] into propionate production instead of CH_4_. In that regard, lactate producers *Sharpea* and *Kandleria* were abundant in the rumens of one of two low CH_4_-producing sheep microbiomes (Kittelmann et al., 2014). Low CH_4_-producing sheep also had higher rumen concentration of lactate, and the lactate dehydrogenases that differed the most between the low- and the high-producing CH_4_ sheep associated phylogenetically with *S. azabuensis* and *K. vitulina* (Kamke et al., 2016). Several strains of *Sharpea* and *Kandleria* that produced predominantly lactate and small amounts of formate, ethanol and acetate, did not change their fermentation products when growing with a methanogen (Kumar et al., 2018).

Lactate produced by *Sharpea* and *Kandleria* did not accumulate to high concentrations in the rumen because it seemed to be metabolized by *Megasphaera* spp. mostly to butyrate, and to propionate via the non-randomizing pathway. The conversion of lactate to butyrate would result in less H_2_ production compared to acetate production from glucose (Kamke et al., 2016). It seems then that a dual mechanism resulted in lower CH_4_ production in the low-CH_4_ producing sheep (Kittelmann et al., 2014; Kamke et al., 2016): (i) Incorporation of [H] in the reduction of pyruvate to lactate by *Sharpea* and *Kandleria* instead of H_2_ release, and (ii) Uptake and conversion of lactate to butyrate and propionate by *Megasphaera*. In this regard, co-culture experiments comparing the kinetics of uptake of lactate and conversion to acetate, propionate and butyrate by *Megasphaera* and other microorganisms would be of interest. *M. elsdenii* had a lower affinity for lactate than for glucose (Russell and Baldwin, 1979), but its rate of lactate uptake was not affected by glucose (Russell and Baldwin, 1978). Interestingly, fermentation extracts of the probiotic *Aspergillus oryzae* stimulated lactate uptake by *M. elsdenii* and did not affect its fermentation profile (Waldrip and Martin, 1993).

This approach toward decreasing CH_4_ formation could thus contemplate the addition of a “microbial team” composed by a lactate producer and a lactate utilizer that metabolizes lactate to propionate or butyrate. Another interesting microbial species could be *Fibrobacter succinogenes*, a fiber degrader which does not produce H_2_ and would thus not contribute [H] to methanogenesis and instead incorporate [H] into succinate production (Morgavi et al., 2010). However, the higher *K*_m_ for H_2_ of *F. succinogenes* compared to methanogens (Asanuma et al., 1999) would imply a lower uptake of H_2_ compared to methanogens at low H_2_ concentration (Figure 4).

**FIGURE 5:**
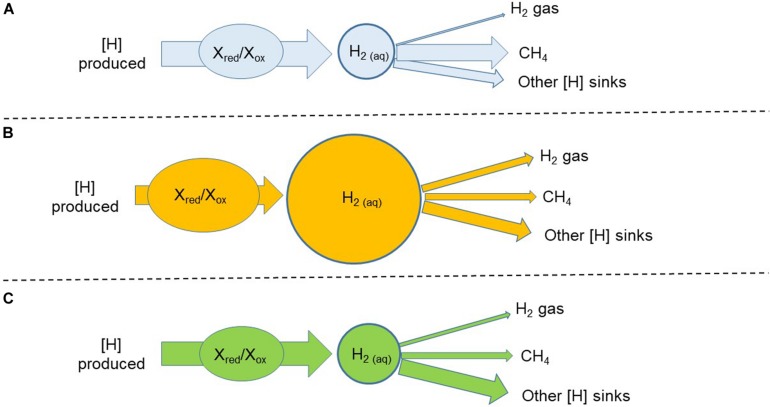
Three hypothetical scenarios of manipulation of metabolic hydrogen ([H]) flows in rumen fermentation: **(A)** Non-intervened rumen fermentation with functional methanogenesis. Methane (CH_4_) is the main sink of metabolic hydrogen; **(B)** Methanogenesis is inhibited with a chemical additive. Part of metabolic hydrogen spared from methane formation is redirected toward alternative sinks that are final fermentation products in the rumen with functional methanogenesis. Redirection of metabolic hydrogen toward alternative sinks is incomplete and the concentration of dissolved dihydrogen increases. The ratio of reduced to oxidized cofactors increases and fermentation, understood as the flow of carbon and the rate of metabolic hydrogen production, is inhibited; **(C)** A theoretical successful situation in which methanogenesis is inhibited with a chemical additive and an added live hydrogenotrophs redirects a greater proportion of metabolic hydrogen toward alternative sinks potentially beneficial to the host animal. Accumulation of dihydrogen is relieved, cofactors can be re-oxidized as in the rumen with functional methanogenesis, and fermentation is not inhibited.

Another strategy would be to inhibit methanogenesis with a chemical compound and simultaneously dose hydrogenotrophs that incorporate H_2_ into a desirable pathway, or pathways. For example, reductive acetogenesis was enhanced in batch cultures through simultaneous inhibition of methanogenesis and addition of reductive acetogens (Nollet et al., 1997; Le Van et al., 1998; Lopez et al., 1999). The kinetic parameters for H_2_ and the H_2_ threshold of the hydrogenotroph of choice would be very important. Compared to the typical rumen fermentation with CH_4_ as the main [H] sink (Figure 5A), inhibiting methanogenesis results in an increase in the incorporation of [H] into alternative sinks, but also in H_2_ as a [H] sink (Figure 5B). Adding a hydrogenotroph with a high *V*_max_ for H_2_ can allow a high flow of H_2_ incorporation into a desirable fermentation product. However, if the *K*_m_ of the added hydrogenotroph for H_2_ were high, H_2_ would still accumulate, and the magnitude of gaseous H_2_ losses could be important. A low *K*_m_ for H_2_ would also result in H_2_ accumulation if the *V*_max_ for H_2_ was low and the rate of fermentation was high, unless the microorganism was dosed in high numbers. Another possibility would be to combine a hydrogenotroph with high *K*_m_ and *V*_max_ for H_2_ with another hydrogenotroph with low *K*_m_ and *V*_max_. A theoretically ideal situation in which dissolved H_2_ concentration and H_2_ emission are at the level of the rumen with functional methanogenesis is depicted in Figure 5C.

A strategy employing chemical inhibitors of methanogenesis to redirect [H] from CH_4_ toward nutritionally useful alternative [H] sinks should evaluate possible direct effects of the chemical inhibitors on non-methanogenic rumen microorganisms, so as to avoid affecting processes such as fiber digestion or propionate production. This aspect cannot be studied in mixed cultures or *in vivo*, because in these systems, changes in non-methanogenic populations can indirectly result from changes in methanogens and CH_4_ production. The potential toxicity of chemical inhibitors of methanogenesis to non-methanogens should instead be studied in pure cultures. Mevastatin and lovastatin inhibited the growth of methanogens but not of major fermentative rumen bacteria, including major fiber degraders and propionate and butyrate producers (Miller and Wolin, 2001). Chloroform at 2 mM inhibited the growth of reductive acetogens and six other rumen bacteria, including fiber degraders and propionate and butyrate producers (Raju, 2016). In contrast, none of the non-methanogenic microorganisms examined were affected by acetylene at 1 mM (aqueous concentration) or 2-bromoethanesulfonate at 10 mM. The effect of *n*-butylisocyanide and 5,5′-dithio-bis-(2-nitrobenzoic acid) on reductive acetogens was concentration- and species-dependent, whilst 1,10-phenanthroline inhibited reductive acetogens at all of the concentrations studied (Raju, 2016). 3-Nitrooxypropanol at 0.1 mM did not inhibit the growth of 11 functionally diverse rumen bacteria or *Escherichia coli*, whilst much lower concentrations inhibited rumen and non-rumen methanogens (Duin et al., 2016).

From an applied point of view, the addition of a hydrogenotroph to the rumen would ideally target post-feeding peaks of dissolved H_2_ with the added microorganism at its exponential phase of growth. This might be difficult if the microbial additive was administered with the feed, as the added microorganism may be at its lag phase of growth at the peak of feed fermentation and H_2_ release in the rumen. If adding hydrogenotrophs at their exponential phase of growth *in vitro* was successful at utilizing H_2_, further developments toward practical application would need to optimize *in vivo* the timing, means of administration, and doses of added hydrogenotrophs.

Recently, Muñoz-Tamayo et al. (2019) conducted a growth and calorimetry experiment with three rumen methanogens. They estimated kinetic, thermodynamic, and growth parameters and predicted that in the long term only one methanogen would survive in tri-cultures. Similar conclusions were reached in another recent theoretical analysis (Lynch et al., 2019). Muñoz-Tamayo et al. (2019) discussed that, as the rumen harbors a diverse community of methanogens, ecological factors such as sensitivity to pH, location in association to fluid or particles, and endosymbiosis with protozoa can contribute to explain the existence of diversity despite of thermodynamic and kinetic advantages of some methanogens over others. Some ecological aspects can cause temporal and spatial variations in H_2_ concentration (Smolenski and Robinson, 1988; Janssen, 2010), which can affect the partition of H_2_ flows among different hydrogenotrophs. In the conceptual model proposed by Leng (2014), the organization of particle-colonizing microbiota in biofilms results in close proximities between cells releasing and taking up H_2_, resulting in ample variations in H_2_ concentration within the rumen.

Greening et al. (2019) reported no differences in the expression of the most abundant H_2_-evolving hydrogenases in sheep selected by low and high CH_4_ production. In contrast, there were differences between low- and high-CH_4_ producing sheep in the expression of H_2_-incorporating hydrogenases. The expression of methanogens hydrogenases and methyl-CoM reductase were lower, and the expression of fumarate reductase and acetyl-CoA synthase (which incorporate H_2_ into propionate production and reductive acetogenesis, respectively) were higher, in the low CH_4_-producing sheep. This can be interpreted as those alternative pathways of [H] incorporation decreasing CH_4_ formation in low CH_4_-producing sheep by competing with [H] with methanogenesis. Alternatively, it can also be interpreted as those pathways of [H] incorporation alternative to methanogenesis becoming upregulated in low CH_4_-producing animals as a response to less CH_4_ production, due perhaps to animal factors such as greater rumen outflow rate or lower rumen pH (Janssen, 2010).

Söllinger et al. (2018) reported that, among all bacterial functional genes, the greatest increase in mRNA abundance occurring 1 h after feeding corresponded to the fumarate reductase subunit C transcript, denoting a stimulation of propionate randomizing pathway associated to peaks of H_2_ emission after feeding. However, despite of the increase in the abundance of fumarate reductase transcripts, H_2_ emissions still increased and propionate concentration did not consistently increase 1 h after feeding. It is possible that the *K*_m_ of H_2_ incorporation into propionate production was relatively high, at least under the conditions of that experiment, which would agree with the higher *K*_m_ for H_2_ of fumarate reducers compared to methanogens reported by Asanuma et al. (1999) in pure cultures.

It should be considered that competition for intercellular e^–^ carriers other than H_2_ (and lactate) also occurs. For example, the *K*_m_ for formate was lower for fumarate reducers compared to methanogens (Asanuma et al., 1999). In the rumen, methanol and methylamines resulting from the metabolism of pectin (Pol and Demeyer, 1988) and betaine and choline (Neill et al., 1978; Mitchell et al., 1979) can be used by methylotrophic methanogens as substrates for CH_4_ production. Importantly, reductive acetogens have also been reported to use methanol and methylamines as [H] donors (Ragsdale and Pierce, 2008; Jeyanathan et al., 2014), including the rumen acetogen *Eubacterium limosum*, a methanol-utilizer (Genthner et al., 1981). It is thought that in the typical rumen fermentation, methanogens drop H_2_ pressure below the threshold for reductive acetogenesis (Ungerfeld and Kohn, 2006). However, it is important to examine both in defined and in mixed cultures the competition between methanogens and reductive acetogens for methanol and methylamines.

### Effects of Dihydrogen Accumulation on the Rates of Fermentation and Digestion

The formation of CH_4_ in the rumen represents an important loss of energy for the animal. Theoretically, inhibiting rumen methanogenesis could divert [H] toward fermentation products with a nutritional value for the animal, and improve animal productivity (Czerkawski and Breckenridge, 1975b; Schulman and Valentino, 1976), although this has not been consistently realized (Ungerfeld, 2018). Compared to the rumen with functional methanogenesis (Figure 5A), inhibiting rumen methanogenesis results in accumulation of H_2_ *in vitro* (Ungerfeld, 2015b) and increased H_2_ emissions *in vivo* (Ungerfeld, 2018), increased ratio of NADH to NAD^+^ (Hino and Russell, 1985; Figure 5B), and decreased reducing potential (Sauer and Teather, 1987). These changes indicate hindering in e^–^ disposal and it is important to understand the consequences that this can have on feed fermentation and digestion. Conceptually, there is little doubt that an imbalance between the rates of reduction and re-oxidation of cofactors can halt fermentation (Wolin et al., 1997), because the turnover rates of cofactors are very high compared to their intracellular concentrations (de Graef et al., 1999). This principle has been experimentally verified as an increase in cellulose degradation when fibrolytic fungi were co-cultured with methanogens or with *S. ruminantium* as hydrogenotrophs (Marvin-Sikkema et al., 1990). The questions are, at which point increases in the ratios of reduced to oxidized cofactors begin to impair fermentation, and how these ratios are in turn affected by H_2_ pressure (Figure 5B). Whether accumulated H_2_ can be re-channeled into other pathways (Figure 5C) has been discussed in the preceding section.

High H_2_ pressure can thermodynamically inhibit NADH oxidoreductases (Gottschalk, 1986). Van Lingen et al. (2016) modeled the effect of H_2_ pressure on the thermodynamic feasibility of NADH oxidation with and without electron confurcation with reduced Fd_red_^2^^–^. With an NAD^+^ to NADH ratio of 3, similar to the NAD^+^ to NADH ratio reported by Hino and Russell (1985) for their control treatments, and in the absence of electron confurcation, NADH oxidation was somewhat under thermodynamic control at H_2_ partial pressures of between 2 × 10^–4^ and 2 × 10^–3^ bar, depending on the intracellular pH (Van Lingen et al., 2016). If rumen headspace H_2_ was to be at equilibrium with dissolved H_2_, the corresponding range of dissolved H_2_ concentrations would be as low as 0.15 to 1.5 μM approximately (calculations not shown), but given the occurrence of H_2_ supersaturation (Wang et al., 2016) it would likely be higher. The same calculation conducted with NADH oxidation occurring through electron confurcation would yield a considerable higher range of dissolved H_2_ concentration between 6 and 100 μM, again assuming equilibrium between gaseous and aqueous H_2_. Therefore, with electron confurcation, the range of dissolved H_2_ concentration at which NADH oxidation becomes thermodynamically controlled coincides or is even higher than previously reported peaks of dissolved H_2_ concentration after feed ingestion, or the dissolved H_2_ concentration reported by Melgar et al. (2019) for methanogenesis inhibition (Table 1). This agrees with the findings by Greening et al. (2019) regarding the importance of confurcating hydrogenases in H_2_ formation in the rumen. The thermodynamic feasibility of NADH oxidation also depends on the intracellular pH (Van Lingen et al., 2016), which in turn depends on the extracellular pH and the bacterial species (Russell, 1991).

Inhibiting methanogenesis *in vitro* results in H_2_ accumulation and consistently inhibits hexoses fermentation as estimated through the stoichiometry of VFA production (Ungerfeld, 2015b). However, the estimation of fermented hexoses from the stoichiometry of VFA production does not consider carbon in fermented hexoses utilized in microbial biomass accretion. In an *in vitro* study with several inhibitors of methanogenesis, no consistent effects on true organic matter digestibility were found, with some decreases but also lack of effects with other additives (Ungerfeld et al., 2019). Effects of inhibiting methanogenesis *in vivo* on digestion and fermentation in the rumen are complex to assess with most animal measurements, as apparent digestibility determinations do not consider microbial biomass and overall tract digestibility could be modified by post-ruminal compensations (Ungerfeld, 2018), and rumen VFA concentrations are affected by, apart from VFA production rates, rates of VFA absorption, passage, incorporation into microbial biomass, and by changes in rumen volume (Dijkstra et al., 1993; Kristensen, 2001; Storm et al., 2012; Hall et al., 2015).

It is of course possible that negative effects of inhibiting methanogenesis on fermentation (Ungerfeld, 2015b) are not caused by H_2_ accumulation *per se*, and instead some of the chemical inhibitors studied could be toxic to microorganisms other than methanogens. An experimental approach to study the effects of H_2_ accumulation on the rate of fermentation without the addition of chemical inhibitors of methanogenesis is the addition of H_2_ gas to the headspace of rumen incubations. In general, adding external H_2_ to rumen cultures has not consistently resulted in an inhibition of fermentation measured as total VFA concentration or apparent digestibility (Schulman and Valentino, 1976; Patra and Yu, 2013; Broudiscou et al., 2014; Qiao et al., 2015). A factor potentially masking the effects of H_2_ gas added to microbial cultures headspace is lack of equilibrium with dissolved H_2_ (Wang et al., 2016). In that regard, two *in vivo* studies in which dissolved H_2_ was indirectly delivered through the reaction of elemental magnesium (Mg) with water, reported decreases in total VFA concentration as a result of the augmented dissolved H_2_ concentration (Wang et al., 2017; Ma et al., 2019), although the limitations of VFA concentration as a metric of rumen fermentation pointed out above are again acknowledged.

Ultimately, if means to efficiently redirect [H] to useful sinks (Figure 5C) could be designed, the extent to which the accumulation of H_2_ can hinder NADH re-oxidation and fermentation would be unimportant from a practical standpoint, as H_2_ would not accumulate when inhibiting methanogenesis (Figure 5C). A perhaps more realistic scenario intermediate between Figures 5B,C in which dissolved H_2_ concentration was only partially relieved, but the rate of digestion and fermentation was not affected, can also be conceived.

Pathways of [H] flow alternative to H_2_ formation can result in the production of other intercellular e^–^ carriers, such as lactate, ethanol, and formate, or final fermentation products such as propionate, all of which also help NADH oxidation (Van Lingen et al., 2016). Formate, succinate or ethanol have been shown to accumulate along with H_2_ when methanogenesis was inhibited *in vitro* (Slyter, 1979; Asanuma et al., 1998; Ungerfeld et al., 2003) and *in vivo* (Martinez-Fernandez et al., 2016, 2017; Melgar et al., 2019), so it is important to understand if the accumulation of those metabolites could potentially inhibit cofactors re-oxidation and fermentation. The effects of lactate, ethanol, formate and propionate on fermentation and digestion are best studied in experiments in which those metabolites are externally added to rumen fermentation as pure compounds. Immig (1996) found that adding formate or succinate to rumen batch cultures did not affect total VFA production. Asanuma et al. (1998) found that added formate was stoichiometrically recovered as CH_4_ and total VFA production was unaffected. Infusion of formic acid into the rumen of sheep did not affect overall tract apparent digestibility (Vercoe and Blaxter, 1965). It is possible that as formate has a high rate of diffusion and is rapidly converted to CH_4_ (Leng, 2014), its accumulation may not affect cofactors re-oxidation and fermentation rate.

Lactate is metabolized to VFA in the rumen (Chen et al., 2019). Even though excess lactate accumulation can inhibit fermentation, this effect would likely be caused by low pH rather than by an impairment of [H] transfer and co-factor re-oxidation. A potential effect of lactate accumulation on fermentation independent of pH would have to be evaluated through, for example, a comparison of the addition of sodium lactate against sodium chloride.

The effects of adding ethanol to rumen cultures was dose-depending, and a decrease in cellulose digestibility occurred with the highest dose (Chalupa et al., 1964), whereas no effects on total VFA concentration was found in another *in vitro* experiment (Pradhan and Hemken, 1970). Emery et al. (1959) found a tendency to decrease OM and N digestibility when feeding ethanol to sheep. However, negative effects of high doses of ethanol on rumen fermentation can be mediated through direct toxicity related to bacterial membranes leakages caused by ethanol (Ingram, 1990), rather than through impairing re-oxidation of cofactors.

As an end product of fermentation, propionate is removed by absorption, passage and incorporation into microbial biomass. The removal of propionate would be thought to occur rapidly enough so as to avoid accumulation causing inhibition of fermentation. In agreement, in several experiments intrarruminal infusion of propionate did not affect overall tract digestibility of various feed fractions (Rook et al., 1963; Sheperd and Combs, 1998; Noziere et al., 2000; Oba and Allen, 2003).

### Effects of Metabolic Hydrogen Flows on Microbial Growth

Flows of [H] in the rumen can affect microbial growth through at least three mechanisms: (i) Variation in the generation of ATP; (ii) Provision of precursors for biosynthesis; (iii) Provision of reducing power. The mechanisms through which this might occur will be developed in this section.

Hydrolysis of ATP is necessary to drive otherwise thermodynamically unfeasible anabolic processes, such as protein synthesis. The rate of ATP generation in fermentation depends on the rate of fermentation, the fermentation profile, and the ATP generated in each fermentation pathway. Acetate production generates ATP through substrate level phosphorylation, the same as propionate non-randomizing pathway. In propionate randomizing pathway, butyrate production, and methanogenesis, ATP is also generated through ETLP (Russell and Wallace, 1997; Hackmann and Firkins, 2015). Production of less reduced fermentation products such as lactate and ethanol generates ATP only in glycolysis and results in less microbial growth per unit of substrate degraded compared to acetate production and methanogenesis (Wolin et al., 1997).

One should bear in mind that increasing ATP generation does not necessarily mean maximizing the “efficiency” of fermentation. As the proportion of the Δ*G* of a pathway coupled to ATP generation increases, the net Δ*G* approaches zero, and the pathway slows down approaching equilibrium. For example, methanogens possessing cytochromes can generate more ATP per mole of CH_4_ produced and have higher growth yields when growing on elevated H_2_ concentration compared to methanogens without cytochromes, but on the other hand they have greater H_2_ thresholds. Methylotrophic methanogens of the order Methanosarcinales have cytochromes and they have evolved to live in environments with low H_2_ concentration by acquiring the capacity of using one carbon compounds as substrates for methanogenesis (Thauer et al., 2008; Vanwonterghem et al., 2016; Lynch et al., 2019).

Organic matter catabolized in the rumen is partitioned into fermentation products i.e., VFA and gases, and microbial biomass. The proportion of carbon in fermented carbohydrates diverted toward microbial cell production increases as the microbial biomass produced per mole of ATP hydrolyzed (*Y*_ATP_) increases (Leng and Nolan, 1984) and as more moles of ATP are generated per mole of hexoses fermented. Also, each fermentation pathway can contribute different intermediate compounds to microbial anabolism.

Microbial biomass is more reduced than substrate fermented, and consequently, alterations in the flows of [H] could affect [H] available for microbial biomass accretion. For example, inhibiting methanogenesis could result in increased [H] disposal into microbial biomass formation (Czerkawski, 1986). Anabolic processes such as amino acids and fatty acids synthesis demand [H] and may be stimulated as a consequence of the inhibition of CH_4_ production (Chalupa, 1977; Ungerfeld, 2015b). Deamination of reduced amino acids was inhibited by reducing power in the form of NADH, and conversely, was stimulated by methylene blue, an oxidizing agent (Hino and Russell, 1985). Later results, however, could not confirm an increase in the incorporation of [H] into microbial amino acids when methanogenesis was inhibited *in vitro* (Ungerfeld et al., 2019).

## Conclusion and Future Directions

Early work in the past century established the foundations to understand fermentation and [H] dynamics in the rumen. Hungate (1967) demonstrated the central role of H_2_ in CH_4_ production. The principles and importance of interspecies H_2_ transfer was illustrated in several ingenious experiments in which H_2_ producers were co-cultured with methanogens (Wolin et al., 1997). A model to explain how the diet influences the VFA profile and CH_4_ formation through changes in methanogens rate of growth and H_2_ concentration (Janssen, 2010) has been an important advancement in this area. Electron confurcation has been incorporated into rumen fermentation models (Van Lingen et al., 2016, 2019), and recent experimental work with comparative genomics and metatranscriptomics revealed the importance of electron bifurcation and confurcation in H_2_ dynamics in the rumen. Shifts in fermentation in defined cultures were studied at the level of gene expression (Greening et al., 2019).

In comparison, fewer studies (Chen and Wolin, 1977; Latham and Wolin, 1977) have examined the competition for H_2_ between methanogens and other hydrogenotrophs, such as succinate and propionate producers, at the basal H_2_ concentrations resulting from fermentation-evolving H_2_. A recent experiment studied the competition for [H] between a methanogen and lactate producers *Sharpea* and *Kandleria* (Kumar et al., 2018). Pure cultures of rumen hydrogenotrophs, such as those isolated in the Hungate 1000 Project (Kelly, 2016), could be screened for kinetic parameters of H_2_ incorporation and H_2_ thresholds. This information could be used to predict the outcome of the competition for H_2_ between methanogens and other hydrogenotrophs with models similar to the ones generated by Muñoz-Tamayo et al. (2019) and Lynch et al. (2019) for competition between methanogens. The ability of non-methanogenic hydrogenotrophs to compete for H_2_ could be evaluated in co-culture with methanogens if they are also fermentative H_2_ producers themselves (e.g., *R. flavefaciens*, *S. ruminantium*), or in tri-cultures with methanogens and a H_2_-producing organism if they incorporate H_2_ but do not produce it (e.g., *F. succinogenes*, *Succinivibrio dextrinosolvens*, *Succinimonas amylolytica*, reductive acetogens).

Understanding the physicochemical control of rumen fermentation can help optimizing the design of strategies to direct [H] from CH_4_ to other sinks. In this regard, H_2_ concentration is highly influential on the thermodynamics and kinetics of fermentation pathways (Janssen, 2010). Research is needed on dissolved H_2_ concentration (Table 1) and H_2_ gradients under different conditions, especially when methanogenesis is inhibited. Dissolved H_2_ concentration has been generally estimated by measuring the concentration of H_2_ gas in the gas phase and assuming equilibrium with dissolved H_2_ in the fluid (Kohn and Boston, 2000; Ungerfeld and Kohn, 2006; Janssen, 2010), but H_2_ has been shown to be supersaturated in the rumen (Wang et al., 2016). It would be important to incorporate H_2_ supersaturation factors in future models of rumen fermentation, but more results with different diets, time after feeding, and other factors such as methanogenesis inhibition, are needed so that H_2_ supersaturation is not modeled as a constant.

The application of genomics and transcriptomics has advanced our understanding of the relationships between the abundance and expression of genes encoding for hydrogenases and rumen [H] flows (Greening et al., 2019). The combination of –omics techniques with classical biochemistry and microbiology methods may make possible the isolation and kinetic characterization of H_2_-incorporating hydrogenases. The application of proteomics to understand methanogenesis and flows of [H] through changes in hydrogenases and other enzymes involved in [H] transactions is also of much interest (Snelling and Wallace, 2017). Recently, metabolomics has been applied toward the understanding of differences between dairy cows with high and low feed utilization efficiency associated to high and low CH_4_ production (Shabat et al., 2016) and toward understanding the responses to methanogenesis inhibitors (Martinez-Fernandez et al., 2018). Finally, experimental advances must be interpreted in the light of basic physicochemical knowledge of thermodynamics and kinetics to develop mathematical and conceptual mechanistic models (Janssen, 2010; Van Lingen et al., 2016) for designing new strategies of manipulation of [H] flows in the rumen and predicting their outcomes.

## Author Contributions

EU reviewed and conceptualized the ideas, and wrote, edited and submitted the manuscript.

## Conflict of Interest

The author declares that the research was conducted in the absence of any commercial or financial relationships that could be construed as a potential conflict of interest.
