# Supplementary material for: Metabolic Hydrogen Flows in Rumen Fermentation: Principles and Possibilities of Interventions
Source: Front Microbiol. 2020 Apr 15;11:589. doi: 10.3389/fmicb.2020.00589 (PMC7174568; doi:10.3389/fmicb.2020.00589)
Supplement: Supplementary file 1 [file Table_1.docx]

Supplementary Material

**Appendix 1**

$$\frac{v\text{met}}{(v\text{met} + v\text{alt})}\boldsymbol{=}\frac{\frac{V\text{max met} [H\text{2}]}{(K\text{m met} + [H\text{2}])}}{\left[ \frac{V\text{max met} [H\text{2}]}{(K\text{m met} + [H\text{2}])}\boldsymbol{+}\frac{V\text{max alt} [H\text{2}]}{(K\text{m alt} + [H\text{2}])} \right]}$$

$$\frac{v\text{met}}{(v\text{met} + v\text{alt})}\boldsymbol{=}\frac{\frac{V\text{max met} [H\text{2}]}{(K\text{m met} + [H\text{2}])}}{\left[ \frac{V\text{max met} \left[ H\text{2} \right]\left( K\text{m alt} + \left[ H\text{2} \right] \right) + V\text{max alt} [H\text{2}](K\text{m met} + [H\text{2}])}{(K\text{m met} + [H\text{2}])(K\text{m alt} + [H\text{2}])} \right]}$$

$$\frac{v\text{met}}{(v\text{met} + v\text{alt})}\boldsymbol{=}\frac{V\text{max met} [H\text{2}](K\text{m alt} + [H\text{2}])}{\left[ V\text{max met} \left[ H\text{2} \right]\left( K\text{m alt} + \left[ H\text{2} \right] \right) + V\text{max alt} [H\text{2}](K\text{m met} + [H\text{2}]) \right]}$$

$$\frac{v\text{met}}{(v\text{met} + v\text{alt})}\boldsymbol{=}\frac{V\text{max met} (K\text{m alt} + [H\text{2}])}{\left[ V\text{max met} \left( K\text{m alt} + \left[ H\text{2} \right] \right) + V\text{max alt} (K\text{m met} + [H\text{2}]) \right]}$$

**Appendix 2**

If *V_max met_* = *V_max alt_* = *V_max_*

$$\lim_{\left[ H2 \right] \to+\infty} \left[ \frac{v\text{met}}{(v\text{met} + v\text{alt})} \right]= \lim_{\left[ H2 \right] \to+\infty}\left\{ \frac{V\text{max met} (K\text{m alt} + [H\text{2}])}{\left[ V\text{max met} \left( K\text{m alt} + \left[ H\text{2} \right] \right) + V\text{max alt} (K\text{m met} + [H\text{2}]) \right]} \right\}$$

$$\lim_{\left[ H2 \right] \to+\infty} \left[ \frac{v\text{met}}{(v\text{met} + v\text{alt})} \right]= \lim_{\left[ H2 \right] \to+\infty}\left\{ \frac{V\text{max} (K\text{m alt} + [H\text{2}])}{\left[ V\text{max } \left( K\text{m alt} + \left[ H\text{2} \right] \right) + V\text{max } (K\text{m met} + [H\text{2}]) \right]} \right\}$$

$$\lim_{\left[ H2 \right] \to+\infty} \left[ \frac{v\text{met}}{\left( v\text{met} + v\text{alt} \right)} \right]= \lim_{\left[ H2 \right] \to+\infty} \left\{ \frac{V\text{max } \left( K\text{m alt} + \left[ H\text{2} \right] \right)}{\left[ V\text{max }\left( K\text{m alt} + \left[ H\text{2} \right]+ K\text{m met} + \left[ H\text{2} \right] \right) \right]} \right\}$$

$$= \lim_{\left[ H2 \right] \to+\infty} \left\{ \frac{\left( K\text{m alt} + \left[ H\text{2} \right] \right)}{\left[ \left( K\text{m alt} + K\text{m met} + 2 \left[ H\text{2} \right] \right) \right]} \right\}\boldsymbol{= ½}$$

**Appendix 3**

If *V_max met_* = ½ *V_max alt_*

$$\lim_{\left[ H2 \right] \to+\infty} \left[ \frac{v\text{met}}{(v\text{met} + v\text{alt})} \right]= \lim_{\left[ H2 \right] \to+\infty}\left\{ \frac{V\text{max met} (K\text{m alt} + [H\text{2}])}{\left[ V\text{max met} \left( K\text{m alt} + \left[ H\text{2} \right] \right) + V\text{max alt} (K\text{m met} + [H\text{2}]) \right]} \right\}$$

$$\lim_{\left[ H2 \right] \to+\infty} \left[ \frac{v\text{met}}{(v\text{met} + v\text{alt})} \right]= \lim_{\left[ H2 \right] \to+\infty}\left\{ \frac{V\text{max met} (K\text{m alt} + [H\text{2}])}{\left[ V\text{max met} \left( K\text{m alt} + \left[ H\text{2} \right] \right) + 2 V\text{max met } (K\text{m met} + [H\text{2}]) \right]} \right\}$$

$$\lim_{\left[ H2 \right] \to+\infty} \left[ \frac{v\text{met}}{(v\text{met} + v\text{alt})} \right]= \lim_{\left[ H2 \right] \to+\infty}\left\{ \frac{V\text{max met} (K\text{m alt} + [H\text{2}])}{V\text{max met} \left[ K\text{m alt} + \left[ H\text{2} \right]+ 2\text{ }K\text{m met} + 2 [H\text{2}]) \right]} \right\}$$

$$\lim_{\left[ H2 \right] \to+\infty} \left[ \frac{v\text{met}}{(v\text{met} + v\text{alt})} \right]= \lim_{\left[ H2 \right] \to+\infty}\left\{ \frac{(K\text{m alt} + [H\text{2}])}{\left[ K\text{m alt } +2\text{ }K\text{m met }+ 3 [H\text{2}]) \right]} \right\}\boldsymbol{= ⅓}$$

**Appendix 4**

Given *m* different methanogens and *n* total different hydrogenotrophs, including methanogens:

$$\frac{\sum_{j=1}^{j=m} v\text{met}j}{\sum_{i=1}^{i=n} vi}= \frac{\sum_{j=1}^{j=m} \frac{vmax metj [H2]}{(Km metj+\left[ H2 \right])}}{\sum_{i=1}^{i=n} \frac{vmaxi [H2]}{(Kmi+\left[ H2 \right])}}$$

$$\frac{\sum_{j=1}^{j=m} v\text{met}j}{\sum_{i=1}^{i=n} vi}= \frac{[H2] \sum_{j=1}^{j=m} \frac{vmax metj}{(Km metj+\left[ H2 \right])}}{\left[ H2 \right] \sum_{i=1}^{i=n} \frac{vmaxi}{(Kmi+\left[ H2 \right])}}$$

$$\frac{\sum_{j=1}^{j=m} v\text{met}j}{\sum_{i=1}^{i=n} vi}= \frac{\sum_{j=1}^{j=m} \frac{vmax metj}{(Km metj+\left[ H2 \right])}}{\frac{\sum_{i=1}^{i=n} vmaxi (Kmi+\left[ H2 \right])}{\prod_{i=1}^{i=n} (Kmi+\left[ H2 \right])}}$$

$$\frac{\sum_{j=1}^{j=m} v\text{met}\text{j}}{\sum_{i=1}^{i=n} vi}= \frac{\prod_{i=1}^{i=n} (Kmi+\left[ H2 \right])\sum_{j=1}^{j=m} \frac{vmax metj}{(Km metj+\left[ H2 \right])}}{\sum_{i=1}^{i=n} vmaxi (Kmi+\left[ H2 \right])}$$
